# Mercury by Inhalation

**Published:** 1851-06

**Authors:** H. J. Richards

**Affiliations:** Philadelphia


*Mercury by Inhalation.* By H. J. RICHARDS, M. D., Philadelphia.

The following curious fact was communicated to me in conversation recently, and has seemed to me worthy of publication. The recent practice of M. Ricord,—rather the testimony given in his late work, of the efficiency of mercury by *fumigation*—the favor with which it was regarded by Mr. Abernethy, associated with this Indian testimony, all point to a possibility that these methods of exhibition may develope more properties in this important medicinal substance; besides furnishing us with a resource in the method of its administration, that may find its occasion.

I have been informed by a gentleman of intelligence, who has resided for a long time in China and Manilla, that mercury, which is there regarded as a specific in the severe forms of hepatic disorder incident to the climate, and leading to its employment to an extent that would be considered extravagant by our European notions, is constantly exhibited in the following novel and peculiar manner. The black oxide is introduced into the manilla cheroots, and being inhaled, is thus presented in the form of vapor to the most absorbent surface in the body, speedily manifesting its constitutional effect. The only difficulty existing is the inability of the physician to regulate the amount of effect. By intelligence on the part of the patient, however, this is obviated; whereas its advantages are such as to obtain for it the preference, in many cases, over the ordinary method. *It certainly is a speedy plan of producing salivation.* I mention this fact as it is interesting in itself, and may suggest other applications of the same principle.

